# Economic evaluation of trimetazidine in the management of chronic stable angina in Greece

**DOI:** 10.1186/s12913-016-1779-6

**Published:** 2016-09-27

**Authors:** Georgia Kourlaba, George Gourzoulidis, George Andrikopoulos, Konstantinos Tsioufis, Alexandra Beletsi, Nikos Maniadakis

**Affiliations:** 1 The Stavros Niarchos Foundation-Collaborative Center for Clinical Epidemiology and Outcomes Research (CLEO), National and Kapodistrian University of Athens, School of Medicine, Thivon & Papadiamantopoulou, Athens, 115 27 Greece; 2 Department of Health Services Organization & Management, National School of Public Health, Athens, Greece; 3 Cardiology Department, Henry Dunant Hospital Center, Athens, Greece; 4 Hippokration General Hospital, First Cardiology Clinic, University of Athens, Athens, Greece; 5 Servier Hellas, Athens, Greece

**Keywords:** Coronary disease, Cost-utility, Vastarel, Angina pectoris

## Abstract

**Background:**

To evaluate the cost-effectiveness of trimetazidine (TMZ) as add-on therapy to standard-of-care (SoC) compared to SoC alone in patients with chronic stable angina who did not respond adequately to first line therapy with b-blockers, nitrates or calcium channel antagonists in Greece.

**Methods:**

A Markov model with 3-month cycles and 1-year time horizon was developed to assess the comparators. The analysis was conducted from a third-party payer perspective. The clinical inputs and utility values were extracted from the published literature. Resource consumption data were obtained from local experts, using a questionnaire developed for the purpose of the study and were combined with unit cost data (in €2016) obtained from official sources. Cost effectiveness was assessed by calculating the incremental cost effectiveness ratio (ICER). Probabilistic sensitivity analysis (PSA) was performed to account for uncertainty and variation in the input parameters of the model.

**Results:**

The analysis showed that the cost of TMZ plus SoC was €1755.57 versus €1751.76 of SoC alone. In terms of health outcomes, TMZ plus SoC was associated with 0.6650 QALYs versus 0.6562 QALYs for SoC alone. The incremental analysis resulted in an ICER of €430.67 per QALY gained. PSA revealed that the probability of TMZ plus SoC being cost-effective over SoC was 89 %, at a threshold of €34,000 per QALY gained.

**Conclusion:**

The results indicate that TMZ as add –on treatment may be a highly cost-effective option for the symptomatic treatment of patients with chronic stable angina in Greece relative to SoC alone.

**Electronic supplementary material:**

The online version of this article (doi:10.1186/s12913-016-1779-6) contains supplementary material, which is available to authorized users.

## Background

Chronic stable angina is a clinical syndrome of temporary hypoxic ischemic status caused mainly by persistent coronary stenosis induced heart load increase [1]. There is increased body of evidence indicating that chronic stable angina strongly affects patient’s quality of life, and patients with chronic stable angina are more likely to have depression [2, 3] or impaired sexual functioning [4] and insomnia compared to the general population. Moreover, there are indications that patients with moderate or severe angina have more than a twofold higher mortality risk compared to those with minimal or mild angina [5].

Apart from the humanistic burden, chronic stable angina may incur great economic consequences for the payers health care systems [6] and society overall, as this condition leads to higher healthcare resource utilization, such as medication and hospitalizations, including in many occasions expensive vascular interventions and loss of productive time. More specifically, hospitalizations have been found to be the main cost driver of burden, as they account for almost two thirds of the total chronic stable angina-related health care expenditures, mainly due to the expensive invasive procedures required for the management of patients [6, 7]. Additionally, studies have shown that chronic stable angina leads to substantial productivity loss [8]. In this light, studies have revealed that the total cost (both direct and indirect) of chronic angina is 2 to 3 times higher compared to the direct chronic angina cost alone [8].

In this context, the effective management of chronic stable angina is paramount for clinical and economic reasons. The aim of patient management in chronic stable angina is to relieve symptoms, to improve quality of life and to prevent cardiovascular events. Lifestyle modification, pharmacological therapy and revascularization procedures represent the cornerstones in management of chronic stable angina [9–11].

The usual pharmacological therapy utilized includes nitrates, beta-blockers, calcium channel blockers, antiplatelets, statins, renin-angiotensin-aldosterone system blockers etc [9–12]. Despite the aggressive use of conventional antianginal therapies, many patients remain symptomatic [13]. For this reason, new classes of treatment with different mechanisms of action have been evaluated recently for the management of chronic stable angina [12, 14–17]. Trimetazidine (TMZ- Vastarel®) is one such novel antianginal agent [18]. TMZ is a pure metabolic agent that induces the myocardium to shift from free fatty acids to predominantly glucose utilization in order to increase adenosine triphosphate (ATP) generation per unit oxygen consumption [19, 20]. In clinical studies TMZ has reduced the frequency of angina episodes and improved exercise performance without affecting hemodynamic parameters [21, 22]. In 2012 the European Medicines Agency (EMA) finished a review of benefits and risks of TMZ and approved its use as add-on therapy for the symptomatic treatment of adult patients with chronic stable angina, who are inadequately controlled by or are intolerant to first-line antianginal therapies [23].

Although it is proven that TMZ represents an effective treatment option for the management of chronic stable angina, it may also impose an additional tangible cost to the health care system and payers. This raises the question as to whether TMZ offers good “value”, in other words a benefit which is worth the investment.

In this light, the aim of the present study was to evaluate the cost-effectiveness of TMZ as add-on therapy to standard-of-care (SoC) compared to SoC alone, in patients with chronic stable angina who did not respond adequately to first line therapy with b-blockers, nitrates or calcium channel antagonists, in the healthcare setting of Greece.

## Methods

A Markov model with 3-month cycles and 1-year time horizon was developed to evaluate the cost-effectiveness of TMZ 35 mg b.i.d as add-on therapy to SoC compared to SoC alone in patients with chronic stable angina. The analysis was conducted from a third-party payer (National Sickness Fund—EOPYY) perspective. Because the time horizon did not exceed 1 year, no discounting was necessary for both cost and health outcomes. The model was set up to calculate the incremental cost incurred and the incremental quality adjusted life years (QALYs) gained using TMZ as add-on treatment for the management of stable angina patients. The cost-effectiveness of TMZ was expressed as an incremental cost effectiveness ratio (ICER). The model was stochastic to allow probabilistic sensitivity analysis.

### Model structure

**Fig. 1:**
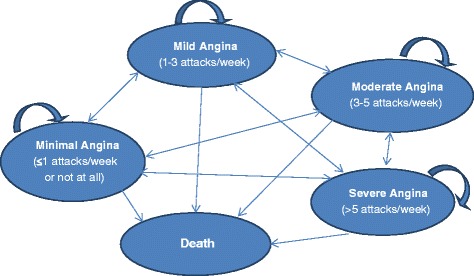
Graphical representation of model structure

The model consists of 5 health states, four of which reflect the severity of chronic stable angina (minimal, mild, moderate, severe) and the other is the absorbing health state of death (Fig. 1). The frequency of angina episodes was used as the main indicator to define angina severity, as follows: 1) minimal angina: episodes occur less than once a week or not at all; 2) mild angina: occur weekly; 3) moderate angina: indicates having symptoms several times per week to every day; and 4) severe angina: reflects having angina several times per day [5, 24].

Patients enter the model in one of the three most severe angina frequency health states (mild, moderate or severe angina) and are assumed to receive TMZ plus SoC or SoC alone. Then, on a cycle basis, patients can transit to one of the other health states, dependent upon specific treatment-related probabilities. For example, patients who enter the model at the mild state may remain in the same state after a 3-month period or move into the minimal, moderate, and severe or death states. Patients may transit from one health state to another during the first 3 months (i.e. 1 cycle), to be in line with the follow-up period of the VASCO trial [25]. In absence of long-term efficacy data for trimetazidine, from the 2nd cycle and onwards, living patients are assumed to remain in the same angina health state for the remainder of the model’s time horizon, or until death.

### Clinical Inputs

The clinical inputs considered in the model were: a) transition probabilities which reflect the probability of patients moving from one health state to another b) the baseline distribution of patients across the health states that reflect the angina severity, and the c) utility values related to angina severity. For the purpose of the present analysis these data were obtained from the VASCO [25] clinical trial and other published studies [5, 26].

### Transition Probabilities

The transition probabilities for patients treated with TMZ 35 mg b.i.d were extracted from the corresponding arm of VASCO trial [25] (Table 1), while the transition probabilities for patients receiving SoC were derived from the comparator arm of the same trial.

**Table 1:** Clinical inputs (3-month probabilities) per therapy arm and severity state

Angina severity state at the end of the 3-month period					
Angina severity state at baseline	Minimal	Mild	Moderate	Severe	Source
TMZ 70 mg/d + SoC					
*Minimal*	94.66 %	4.20 %	1.15 %	0.00 %	VASCO trial [25]
*Mild*	52.72 %	41.30 %	5.43 %	0.54 %	
*Moderate*	19.42 %	61.17 %	15.53 %	3.88 %	
*Severe*	14.29 %	18.10 %	24.76 %	42.86 %	
SoC					
*Minimal*	90.61 %	9.39 %	0.00 %	0.00 %	
*Mild*	46.60 %	41.88 %	9.42 %	2.09 %	
*Moderate*	25.84 %	43.82 %	23.60 %	6.74 %	
*Severe*	11.72 %	26.56 %	15.63 %	46.09 %	

*TMZ* Trimetazidine, *SoC* Standard of Care

Regarding transition probabilities to death, mortality data were modelled to be conditional upon the angina severity health state, but not treatment used, as no mortality data specifically related to TMZ are available. These mortality rates were derived from a prospective cohort study of coronary artery disease patients from 6 Veterans Affairs General Internal Medicine Clinics that reported annual mortality stratified by angina frequency using the SAQ [5]. These data have been already used elsewhere [24]. The annual mortality data were converted to 3-month transition probabilities using the formula (Table 2): P_monthly_ = 1-exp(-(-ln(1-P_12-month_))*1/4).

**Table 2:** Patient distribution, mortality and utility per severity state

Angina severity state	Annual mortality by angina severity state^a^	Patients’ baseline distribution by angina severity state^b^	Utility values^c^
*Minimal*	4.60 %	0 %	0.81
*Mild*	4.80 %	47 %	0.75
*Moderate*	8.10 %	26 %	0.60
*Severe*	10.90 %	27 %	0.39

^a^Spertus et al [5]

^b^VASCO trial [25]

^c^Longworth et al [26]

### Target population

The target population consisted of a hypothetical patient cohort suitable for being treated with both alternatives. In particular, the base case analysis was conducted for symptomatic patients (>1 angina attacks/week). The baseline distribution of patients across the health states was obtained from VASCO trial (Table 2) [25].

### Utility weights

QALYs were calculated by multiplying the time spent in each health state by corresponding EuroQol-5D utility estimates for each angina severity health state. Utility values range from 0 to 1, where 1 represents perfect health and 0 represents death. Utility values were obtained from the literature (Table 2) [26] and have been used previously in a similar study [27].

### Costing Methodology

The economic evaluation was conducted from a third-party-payer perspective and as such only health care costs reimbursed by the payer were considered. Hence other costs, such as those related to the central Government budget to cover personnel salaries or patient copayments, were not considered. The average resource consumption for a Greek patient suffering from chronic stable angina was obtained from two local key opinion leaders (KOLs) in the field of cardiology. The questionnaire developed for the purpose of the study is presented in Additional file 1. Costs considered in the model include hospitalizations, vascular interventions, outpatient management, medication use, and laboratory and diagnostic examinations. All costs refer to the year 2016 (€).

#### Drug acquisition cost of anti-anginal drugs

Drug acquisition costs for TMZ and SoC were calculated using the latest price bulletin issued by the Ministry of Health (31.12.2015) [28] as well as the corresponding reimbursement prices (Positive List for the reimbursement of medicines, Ministry of Health: Official Government Gazzete, FEK 416/19.2.2016). Reimbursement prices were reduced by the patient relevant patient co-payment (25 %) and relevant rebates (Official Government Gazzete, FEK 64/16.1.2014). In particular, the rebate of 9 % given by manufacturers to get into the positive list was considered in the analysis. For TMZ only, an additional rebate of 2 % was considered as it is alone in its cluster (Official Government Gazzete, FEK 416/19.2.2016). It is noted that the volume-related rebate ranging from 2 to 12 % could have been considered in the analysis. Due to lack of volume–related data for all drugs, it was not taken into account in the base case analysis, but scenario analyses were explored in sensitivity analysis.

SoC drug costs have been estimated using the proportion of patients using each therapeutic class, as obtained from data provided by the local KOLs and data extracted from the Greek population enrolled in the CLARIFY study[29]. The mean daily drug dose was also reported by the local KOLs and the relevant drug acquisition costs for each category were calculated as mentioned above. Data obtained from the CLARIFY study and local KOLs were considered more appropriate than the VASCO trial, as they reflect more accurately the common clinical practice in Greece and ensure the applicability of our results to the Greek setting.

For each therapeutic class, the active substances (INN) considered in the analysis reflected the most commonly prescribed in Greece, as obtained from the local KOLs (Additional file 2). Hence, a weighted cost was calculated for each therapeutic class based on the distribution of patients to different active substances. Regarding the drug acquisition cost of TMZ, a daily dose of 35 mg b.i.d, which reflects the common clinical practice in Greece, was considered to be aligned with the dose used in the VASCO trial [25] from which efficacy data were extracted. Based on this approach, the monthly weighted cost to payer for SoC in Greece was calculated at €23.40 and the monthly cost of TMZ 70 mg/d plus SoC at €29.18

#### Hospitalization costs

To estimate the total annual hospitalization cost (excluding hospitalizations related to vascular interventions) per therapy arm, the reimbursed cost per hospitalization to intensive care unit (ICU) and cardiac care clinic were obtained from the Government Gazette (FEK A’3054/18-11-2012) and the Diagnostic Related Groups (DRGs) tariffs issued by the Greek Ministry of Health, [30] respectively. These were multiplied by the proportion of patients requiring hospitalization and the hospitalization rate (frequency of hospitalizations) as obtained from the local KOLs. The annual hospitalization cost was found to range from €5 in patients experiencing minimal angina episodes to €1429 in patients experiencing severe angina episodes (Table 3).

**Table 3:** Medical costs per category and severity stage

Total annual cost per angina severity	Minimal	Mild	Moderate	Severe
*Outpatient visits* ^a^ *(in €)*	8.00	15.75	39.88	76.50
*Diagnostic tests* ^b^ *(in €)*	41.62	62.05	101.00	122.37
*Laboratory tests* ^b^ *(in €)*	20.41	25.63	45.31	61.24
*Hospitalization without revascularization* ^c^ *(in €)*	4.67	35.03	331.06	1428.63
*Hospitalization with revascularization* ^c^ *(in €)*	586.24	683.62	1583.23	1972.77

Official Source

^a^The cost of physician visit was obtained from government gazette (FEK A’262/16-12-2011)

^b^The cost of diagnostic & laboratory tests was obtained from official site of EOPYY

^c^The cost of hospitalization without revascularization such as intensive care unit & cardiac clinic were obtained from government gazette (FEK A’3054/18-11-2012) and Diagnostic Related Groups (DRG) (average cost of DRG code: K32A, K32M, K47M, K47X)

^d^The cost of hospitalization with revascularization such as PCI&CABG were obtained from DRG (average cost of DRG code: K15X, K10M, K15M for PCI and average cost of DRG code: K05M, KO5X for CABG)

Regarding the cost of vascular interventions, the proportion of patients with chronic stable angina undergoing revascularization, such as percutaneous coronary intervention (PCI) or coronary artery bypass grafting (CABG), in clinical practice in Greece, as obtained from local KOLs, was combined with the reimbursed cost per revascularization as obtained from the DRGs tariffs issued by the Greek Ministry of Health [30]. The annual cost of vascular interventions was found to range from €586 in patients experiencing minimal angina episodes to €1973 in patients experiencing severe angina episodes (Table 3). To account for the effect of angina severity on the cost of hospitalizations, the model allowed the proportion of patients requiring hospitalization (with and without revascularization) and the hospitalization frequency to depend upon angina severity.

#### Routine Monitoring costs

The routine monitoring costs reflect outpatient visits, laboratory tests and diagnostic tests undertaken. The number of visits, laboratory tests and diagnostic tests required as well as the proportion of patients undergoing each test were retrieved from local KOLs. The corresponding reimbursed unit costs were obtained from the Government Gazette (FEK A’262/16-12-2011) and from the official site of EOPYY, respectively. The annual cost for outpatient visits was found to range from €8 in patients suffering from minimal angina to €77 in those suffering from severe angina. With respect to laboratory and diagnostic tests, it was found that the annual laboratory test cost varies from €20 to €62 and the annual diagnostic tests cost from €42 to €122 (Table 3). As with the hospitalizations, the model allowed the proportion of patients undergoing to laboratory and diagnostic tests as well as the number of tests and visits to depend on angina severity.

### Analyses

The aforementioned data were used to estimate mean QALY gain and total mean direct costs attributable to each comparator. The cost-effectiveness of TMZ plus SoC compared to SoC alone was evaluated by calculating the incremental cost per QALY gained (ICER). For a treatment to be considered cost-effective a willingness-to-pay (WTP) threshold of €34,000 per QALY was used as a benchmark in the current analysis. This is based on the World Health Organization (WHO) guidelines which indicate that a treatment is highly cost-effective when the ICER is below the annual Gross Domestic Product (GDP) per capita and it remains cost-effective if the ICER is between 1 and 3 times the GDP per capita of the country for which the analysis is undertaken [31]. Due to critical financial situation of the country at present we have set a maximum limit at 2 times the annual GDP per capita, the latter estimated at about €17,000 from the International Monetary Fund (IMF) at current prices [32].

The majority of input data used in the current model are subjected to uncertainty. Probabilistic sensitivity analysis (PSA) was therefore performed using second-order Monte Carlo simulation. In this analysis, probability distribution was assigned around each parameter (i.e. costs—excluding drug acquisition costs-, transition probabilities etc) and the economic and health outcomes were generated multiple times by drawing samples from the distributions. Distributions were selected according to the nature of the variables utilized [33]. In particular, a gamma distribution was used to represent the uncertainty in costs, because costs are constrained on the interval zero to positive infinity and are often highly positively skewed. Resource use data follow discrete Poisson-distributions, whose conjugate distribution to describe the mean are the gamma distribution. Binomial parameters and utility values are constricted on the interval zero to one and hence they were varied according to a beta distribution. For multinomial data such as transition probabilities, a Dirichlet distribution was used. In total, 5000 estimates of costs, QALYs, and ICERs. A cost-effectiveness acceptability curve (CEAC) was plotted, showing the proportion of simulations that are considered cost-effective at different levels of WTP for a QALY.

Finally, to assess the impact of the assumptions considered in the base case analysis, one-way sensitivity analyses (OWSA) was undertaken to test the robustness of the results, by varying individual parameters between low and high values, in order to ascertain the key drivers of cost-effectiveness. The upper and lower bounds of all parameters were set at ±20 and ±10 % for cost and clinical inputs respectively (assumption) of the base case values.

All statistical calculations performed using Microsoft Excel 2010.

## Results

### Deterministic results

The analysis showed that TMZ plus SoC was marginally more costly than Soc alone (€1755.57 versus €1751.76). Among the cost categories considered, vascular interventions accounted for 56 % and 58 % of total costs in TMZ plus SoC and SoC alone, respectively. Following vascular interventions, hospitalization cost (without vascular intervention) made up 17 % and 18 % of the total annual cost related to TMZ plus SoC and SoC alone, respectively, and drug acquisition cost accounted for 19 % and 16 % of the total costs, respectively. The remaining medical costs (i.e. laboratory tests, diagnostic tests and physician visits) represented only a small proportion of overall costs.

In terms of health outcomes, the analysis revealed that TMZ plus SoC was marginally more effective compared to SoC alone. Patients who received TMZ plus SoC gained 0.6650 QALYs while patients who received SoC alone gained 0.6562 QALYs. Under the base case assumptions, incremental analysis showed that TMZ plus SoC was more effective and more costly than SoC resulting in an ICER of €430.67 per QALY gained well below the predetermined WTP threshold (Table 4).

**Table 4:** Deterministic results per therapy arm

	TMZ 70 mg/d + SoC	SoC alone	Incremental
Costs			
Total cost (€)	1755.57	1751.76	3.82
Drug acquisition cost (€)	341.36	273.61	67.75
Hospitalization cost (€)	293.63	320.72	−27.09
Outpatient visits cost (€)	26.89	28.28	−1.39
Vascular interventions cost (€)	990.01	1022.87	−32.87
Diagnostic tests cost (€)	71.22	72.93	−1.71
Laboratory tests cost (€)	32.47	33.35	−0.88
Health Outcomes			
QALYs	0.6650	0.6562	0.0088
LYs	0.9747	0.9743	0.0004
Incremental analysis			
ICER per QALY (€)			430.67

*ICER* Incremental cost effectiveness ratio, *QALYs* Quality-adjusted life years, *LYs* Life years, *TMZ* Trimetazidine, *SoC* Standard of Care

### One-way sensitivity analysis

**Fig. 2:**
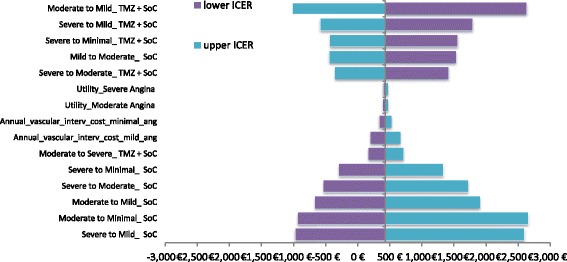
Tornado diagram of one-way sensitivity analysis

The OWSA revealed that the results of the model were more sensitive to the transition probability of moderate to mild angina of patients treated with TMZ plus SoC as this parameter was found to have the greatest effect on ICER, followed by the transition probabilities of severe to mild angina and moderate to mild of patients treated with SoC (Fig. 2). It is worth noting that in all parameters considered in the sensitivity analysis the TMZ plus SoC remains a cost—effective treatment, since the ICER per QALY gained remains well below of the threshold of €34,000 per QALY gained. Moreover, when a volume rebate of 2 and 12 % on reimbursed drug prices of TMZ and SoC was considered in analysis, the TMZ plus SoC was a cost-effective (ICER: €273 per QALY gained) and dominant (less costly, more effective) alternative over SoC respectively.

### Probabilistic sensitivity analysis

**Fig. 3:**
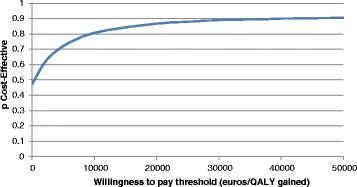
Cost-effectiveness acceptability curve TMZ plus SoC vs SoC alone

The PSA confirms the deterministic results. In particular, the analysis showed that TMZ plus SoC is more cost-effective than SoC for the majority of iterations. The CEAC showed that the likelihood of TMZ plus SoC being cost-effective at a WTP of €34,000/QALY was found to be 89 % compared to SoC alone [Fig. 3].

## Discussion

In the present study, a Markov model was developed to evaluate the cost effectiveness of TMZ as add-on therapy to SoC compared to SoC alone during a 1-year time horizon in patients with chronic stable angina in Greece. The analysis was conducted from a third-party payer perspective such as only direct medical costs were considered in the analysis. To the best of our knowledge, this is the first study aiming to evaluate the cost effectiveness of TMZ as add-on therapy to SoC compared to SoC.

The current analysis showed that TMZ as add-on therapy to SoC was slightly more costly treatment as it was found that the total treatment cost was higher by €3.81 compared to SoC alone over a 1-year time horizon. In terms of health outcomes, TMZ was more effective resulting in an ICER equal to €430.67 per QALY gained, well below the predetermined WTP threshold of €34,000 per QALY gained. This finding seems to be reasonable as the modest increased cost of medicines in TMZ plus SoC arm, was partially offset by expected reductions in hospitalization and vascular interventions costs compared to SoC alone. To examine the robustness of this finding, an OWSA was conducted. TMZ plus SoC remained a cost-effective alternative even when a 20 % increase at the acquisition cost of TMZ was considered in the analysis.

Our findings in combination with the clinical benefits provided by TMZ such as the statistically significantly greater improvement of total exercise duration and time to 1-mm ST segment depression compared to placebo indicates that TMZ is a favorable choice for the management of chronic stable angina compared to placebo. Moreover, it should be noticed that no difference has been identified in serious adverse events between TMZ and placebo indicating that TMZ is well-tolerated [34].

Although the methodology adopted followed standard conventions and various sensitivity analyses were conducted to fully explore uncertainty, several potential limitations to this study should be considered. First of all, in the present analysis it was assumed that the clinical and utility data obtained from the published studies [5, 25, 26] were applicable to the Greek health care setting. The use of this data may be challenged; however, given the absence of local data this choice was unavoidable. One may argue that pivotal trials such as VASCO are almost universally used to build models for pricing and reimbursement decisions. Moreover, it is worth noting that the profile of Greek patients, including smoking habits, co-morbidities, medical history, age and body mass index, as described in the CLARIFY study [29], is comparable with that of participants in VASCO. This finding enhances the application of VASCO results to the Greek population. However, the mean age of Greek patients seems to be lower than that of the cohort of patients from Veterans Affairs General Internal Medicine Clinics from which mortality data was extracted; something that may introduce a bias in our estimations. Nevertheless, in absence of more relevant data, the use this mortality data was unavoidable.

Secondly, in the absence of local data, resource utilization was estimated by local experts. This may raise concerns about the subjectivity of model inputs and leave space for challenging the study results. Nonetheless, the experts participating in the study are well-known cardiologists with extensive clinical experience on the management of chronic stable angina, and were consistent in their views.

Thirdly, the 1-year time horizon sounds very short to assess the cost-effectiveness of a treatment for a chronic condition. However, long-term TMZ efficacy data as well as mortality data are completely lacking from the literature. This is the reason that in the vast majority of studies aiming to evaluate the cost effectiveness of the management of chronic stable angina such a short time horizon is considered. Fourthly, the efficacy of TMZ was applied only in the first 3 months of our analysis assuming that the effect achieved at the end of the 3rd month remains till death or the end of analysis. This assumption may overestimate of underestimate the health benefit related to TMZ used. However, there are no long-term efficacy data for TMZ available, and any extrapolation was not feasible as we did not have any access to the raw data of VASCO trial. At this point, it should be noted that.

## Conclusion

In conclusion, the ICERs obtained in different scenarios of the present study are very much below established benchmarks, and hence the present results indicate that TMZ as add–on treatment is a highly cost-effective alternative for the symptomatic treatment of patients with chronic stable angina in Greece.

## Additional files

Additional file 1: Questionnaire. Data description: The questionnaire used to collect data from local Key Opinion Leaders is presented in the file. (DOCX 65 kb) Additional file 2: Drug acquisition cost. Data description: Proportion of patients using each therapy, mean drug daily dose and relative drug acquisition cost is presented in the file. (DOCX 70 kb)

## Abbreviations

ATP: Adenosine triphosphate
CABG: Coronary artery bypass grafting
CEAC: Cost-effectiveness acceptability curve
DRGs: Diagnostic Related Groups
EMA: European Medicines Agency
GDP: Gross Domestic Product
ICER: Incremental cost effectiveness ratio
ICU: Intensive care unit
IMF: International Monetary Fund
INN: Active substances
KOLs: Local key opinion leaders
OWSA: One-way sensitivity analysis
PCI: Percutaneous coronary intervention
PSA: Probabilistic sensitivity analysis
QALYs: Quality adjusted life years
SoC: Standard-of-care
TMZ: Trimetazidine
WHO: World Health Organization
WTP: Willingness-to-pay
