# Supplementary material for: Economic evaluation of trimetazidine in the management of chronic stable angina in Greece
Source: BMC Health Serv Res. 2016 Sep 27;16:520. doi: 10.1186/s12913-016-1779-6 (PMC5039874; doi:10.1186/s12913-016-1779-6)
Supplement: Additional file 1: — Questionnaire. Data description: The questionnaire used to collect data from local Key Opinion Leaders is presented in the file. (DOCX 65 kb) [file 12913_2016_1779_MOESM1_ESM.docx]

**Additional file 1**: The questionnaire used for data collection

| **Α.** **Medication** | | | | |
| --- | --- | --- | --- | --- |
| **Please provide the proportion of patients (pts) with stable angina who receive medical treatment per therapeutic class. Moreover, please provide the most commonly prescribed active substances (INN) per therapeutic class, the mean daily dose and the proportion of patients which receives each INN within the therapeutic class.** | | | | |
| **Therapeutic class** | **%**  **Of pts per therapeutic class** | **Active substance** | **Daily dose (dose x frequency)** | **% of pts within each therapeutic class*** |
| **statins** |  |  |  |  |
|  |  |  |  |  |
|  |  |  |  |  |
|  |  |  |  |  |
| **b-blockers** |  |  |  |  |
|  |  |  |  |  |
|  |  |  |  |  |
| **ACE Inhibitors** |  |  |  |  |
|  |  |  |  |  |
|  |  |  |  |  |
|  |  |  |  |  |
| **Angiotensin II receptot blockers** |  |  |  |  |
|  |  |  |  |  |
|  |  |  |  |  |
|  |  |  |  |  |
| **Long-acting nitrates** |  |  |  |  |
|  |  |  |  |  |
|  |  |  |  |  |
|  |  |  |  |  |
| **Ca-blockers** |  |  |  |  |
|  |  |  |  |  |
|  |  |  |  |  |
|  |  |  |  |  |
| **Anticoagulants** |  |  |  |  |
|  |  |  |  |  |
|  |  |  |  |  |
|  |  |  |  |  |
| **Antiplatelets** |  |  |  |  |
|  |  |  |  |  |
|  |  |  |  |  |
|  |  |  |  |  |

*percentages within each therapeutic class should be sum to 100%

| **Β. Hospitalization** | | | | | | | |
| --- | --- | --- | --- | --- | --- | --- | --- |
| **B.1 Please provide the proportion of pts who requires hospitalization (either in intensive care unit (ICU) or in cardiac clinic) during a one-year period (excluding vascular interventions), the proportion of patients hospitalized in ICU and the proportion of patients hospitalized in cardiac clinic*. Additionally, please provide the number of hospitalizations in ICU and cardiac clinic, separately, within a one-year period.** | | | | | | | |
| **Angina severity** | **% of pts which required hospitalization either in ICU or in cardiac clinic, during a 1-year period** | **% of pts hospitalized in ICU during a one-year period** | | **Number of hospitalizations in ICU during a one-year period** | **% of pts hospitalized in cardiac clinic during a one- year period** | | **Number of hospitalizations in cardiac clinic during a one-year period** |
| Minimal |  |  | |  |  | |  |
| Mild |  |  | |  |  | |  |
| Moderate |  |  | |  |  | |  |
| Severe |  |  | |  |  | |  |
| **Β2. Please provide the proportion of pts who requires revascularization such as CABG, PCI and stent, dependent on the severity of angina, during a one-year period.** | | | | | | | |
|  | **% CABG** | | **% PCI** | | | **% stent** | |
| Minimal |  | |  | | |  | |
| Mild |  | |  | | |  | |
| Moderate |  | |  | | |  | |
| Severe |  | |  | | |  | |

*% of pts hospitalized in ICUs +% of pts hospitalized in cardiac clinics≥100%

| C. Management of patients with stable angina in outpatient setting | | |
| --- | --- | --- |
| **C.1Please provide the proportion of pts who visits physicians at their private office, as well as the number of visits during a one-year period.** | | |
| **Angina severity** | **Number of visits** | **(%)**  **Pts** |
| Minimal |  |  |
| Mild |  |  |
| Moderate |  |  |
| Severe |  |  |

| **C.2 Please provide the proportion of pts who are subjected to diagnostic and laboratory tests, as well as the number of these tests during a one-year period.** | | | | | | | | |
| --- | --- | --- | --- | --- | --- | --- | --- | --- |
| **Diagnostic tests** | **Angina severity** | | | | | | | |
|  | **Minimal** | | **Mild** | | **Moderate** | | **Severe** | |
|  | **Number of tests** | **(%)**  **Pts** | **Number of tests** | **(%)**  **Pts** | **Number of tests** | **(%)**  **Pts** | **Number of tests** | **(%)**  **Pts** |
| ECG |  |  |  |  |  |  |  |  |
| Treadmill Stress test |  |  |  |  |  |  |  |  |
| Thallium Scintigraphy |  |  |  |  |  |  |  |  |
| Stephaniography |  |  |  |  |  |  |  |  |
| Stress Echo |  |  |  |  |  |  |  |  |
| Chest x-ray |  |  |  |  |  |  |  |  |
| CT stephaniography |  |  |  |  |  |  |  |  |
| Transthoracic echocardiogram |  |  |  |  |  |  |  |  |
| Other |  |  |  |  |  |  |  |  |
| **Laboratory Tests** | **Minimal** | | **Mild** | | **Moderate** | | **Severe** | |
|  | **Number of tests** | **(%)**  **Pts** | **Number of tests** | **(%)**  **Pts** | **Number of tests** | **(%)**  **Pts** | **Number of tests** | **(%)**  **Pts** |
| Complete Blood tests |  |  |  |  |  |  |  |  |
| GLU |  |  |  |  |  |  |  |  |
| CK-MB |  |  |  |  |  |  |  |  |
| Troponin test (TnT) |  |  |  |  |  |  |  |  |
| SGOT/AST |  |  |  |  |  |  |  |  |
| SGPT/ALT |  |  |  |  |  |  |  |  |
| CPK/CK |  |  |  |  |  |  |  |  |
| LDH |  |  |  |  |  |  |  |  |
| Other |  |  |  |  |  |  |  |  |
|  |  |  |  |  |  |  |  |  |
