# Supplementary material for: Economic evaluation of trimetazidine in the management of chronic stable angina in Greece
Source: BMC Health Serv Res. 2016 Sep 27;16:520. doi: 10.1186/s12913-016-1779-6 (PMC5039874; doi:10.1186/s12913-016-1779-6)
Supplement: Additional file 2: — Drug acquisition cost. Data description: Proportion of patients using each therapy, mean drug daily dose and relative drug acquisition cost is presented in the file. (DOCX 70 kb) [file 12913_2016_1779_MOESM2_ESM.docx]

Drug acquisition cost

| **Therapeutic Class** | **Most commonly prescribed INN** | **% of pts¶** | **Daily Dose (mg)‡** | **Drug acquisition Cost/mg (in €)^§^** | **Total drug acquisition cost/month (in €)** |
| --- | --- | --- | --- | --- | --- |
| **Statins** | **Combined** | **90.98%** |  |  | **5.34** |
|  | Atorvastatin | 50% | 20 | 0.0090 | 2.75 |
|  | Simvastatin | 30% | 20 | 0.0125 | 2.28 |
|  | Rosuvastatin | 20% | 10 | 0.0138 | 0.84 |
| **b-blockers** | **Combined** | **82.12%** |  |  | **0.77** |
|  | Bisoprolol | 20% | 5 | 0.0111 | 0.34 |
|  | Metoprolol | 60% | 63 | 0.0004 | 0.40 |
|  | Atenolol | 20% | 38 | 0.0009 | 0.20 |
| **ACE Inhibitors** | **Combined** | **60.52%** |  |  | **2.05** |
|  | Perindopril | 30% | 5 | 0.0211 | 0.96 |
|  | Ramipril | 30% | 5 | 0.0388 | 1.78 |
|  | Enalapril | 30% | 10 | 0.0071 | 0.65 |
| **Angiotensin II receptot blockers** | **Combined** | **70.52%** |  |  | **3.68** |
|  | Valsartan | 50% | 160 | 0.0010 | 2.47 |
|  | Irbesartan | 40% | 150 | 0.0010 | 1.78 |
|  | Candesartan | 10% | 16 | 0.0185 | 0.90 |
| **Ca-blockers** | **Combined** | **54.23%** |  |  | **2.39** |
|  | Amlodipine | 60% | 5 | 0.0252 | 2.31 |
|  | Diltiazem | 40% | 200 | 0.0009 | 2.10 |
| **Long acting nitrates** | **Combined** | **51.05%** |  |  | **0.12** |
|  | Isosorbide Dinitrate | 90% | 5 | 0.0016 | 0.23 |
| **Antiplatelets** | **Combined** | **97.5%** |  |  | **9.06** |
|  | Acetylsalicylic acid | 85% | 100 | 0.0004 | 1.02 |
|  | Clopidogrel | 70% | 75 | 0.0052 | 8.27 |
| **Anticoagulants** | **Combined** | **9.03%** |  |  | **0.11** |
|  | Acenocoumarol (Coumaric) | 100% | 2 | 0.0202 | 1.23 |
| **TMZ** |  |  | 70 | 0.0027 | **5.78** |
| ¶pts: patients,TMZ: Trimetazidine  **Official Source**: Based on local experts and from the study “Clinical Presentation and Management of Stable Coronary Artery Disease: Insights from the International Prospective CLARIFY Registry – Results from the Greek National Cohort” ^[^[^29^](#_ENREF_29)^]^  **‡ Official Source**: Based on local experts  **§ Official Source:** latest price bulleting issued by the Ministry of Health (31.12.2015) ^[^[^28^](#_ENREF_28)^]^ corresponding reimbursement prices (Positive List for the reimbursement of medicines, Ministry of Health: Official Government Gazzete, FEK 416/19.2.2016) | | | | | |
